# Sex-Specific Effects of Testosterone on the Sexually Dimorphic Transcriptome and Epigenome of Embryonic Neural Stem/Progenitor Cells

**DOI:** 10.1038/srep36916

**Published:** 2016-11-15

**Authors:** Matthew S. Bramble, Lara Roach, Allen Lipson, Neerja Vashist, Ascia Eskin, Tuck Ngun, Jason E. Gosschalk, Steven Klein, Hayk Barseghyan, Valerie A. Arboleda, Eric Vilain

**Affiliations:** 1 Department of Human Genetics, David Geffen School of Medicine, University of California Los Angeles, Los Angeles, 90095, CA, USA; 2 Department of Pathology and Laboratory Medicine, David Geffen School of Medicine, University of California Los Angeles, Los Angeles, 90095, CA, USA.

## Abstract

The mechanisms by which sex differences in the mammalian brain arise are poorly understood, but are influenced by a combination of underlying genetic differences and gonadal hormone exposure. Using a mouse embryonic neural stem cell (eNSC) model to understand early events contributing to sexually dimorphic brain development, we identified novel interactions between chromosomal sex and hormonal exposure that are instrumental to early brain sex differences. RNA-sequencing identified 103 transcripts that were differentially expressed between XX and XY eNSCs at baseline (FDR = 0.10). Treatment with testosterone-propionate (TP) reveals sex-specific gene expression changes, causing 2854 and 792 transcripts to become differentially expressed on XX and XY genetic backgrounds respectively. Within the TP responsive transcripts, there was enrichment for genes which function as epigenetic regulators that affect both histone modifications and DNA methylation patterning. We observed that TP caused a global decrease in 5-methylcytosine abundance in both sexes, a transmissible effect that was maintained in cellular progeny. Additionally, we determined that TP was associated with residue-specific alterations in acetylation of histone tails. These findings highlight an unknown component of androgen action on cells within the developmental CNS, and contribute to a novel mechanism of action by which early hormonal organization is initiated and maintained.

Pivotal studies on the rodent developing brain led to the organizational-activational hypothesis, which states that exposure to gonadal hormones are a strong contributing factor in the early development of the sexually dimorphic male and female brain^1^. The role of gonadal hormones on sexual differentiation, and brain masculinization has been investigated in the rodent model over the past century, and has been comprehensively reviewed elsewhere^2^,^3^. Despite extensive investigation looking at the direct effects of chromosome complement^4^,^5^,^6^,^7^, gonadal hormones and epigenetic influences^8^,^9^,^10^,^11^,^12^ on the developing brain, there is a paucity of information that has addressed the multifaceted role of these factors on the progenitor cells that generate the central nervous system—neural stem cells (NSCs). To date, sex differences in NSCs have been limited to showing that sexual dimorphisms exist in the protein expression of P450 Aromatase (CYP19A1) in adult rat and mouse NSCs isolated from the sub-ventricular zone (SVZ)^13^,^14^. This enzyme is responsible for the conversion of testosterone derivatives into active estrogens and is associated with rodent brain masculinization^15^. Furthermore, expression of P450 Aromatase correlates with differences in cellular proliferation, and differentiation^13^,^14^. In addition to sexual dimorphisms, NSCs have also been shown to respond to gonadal hormones in a developmentally and/or site specific manner. For example, 19-Nortestosterone, and 17β Estradiol can negatively regulate the proliferation of NSCs derived from the lateral ventricles of adult rat brains, whereas 17β Estradiol exposure on embryonic derived rat NSCs seem to have a positive regulatory effect, as well as increasing neurogenesis^16^,^17^. These studies have established that neural stem cells respond to gonadal hormones, albeit in different ways depending on either developmental time and/or site of isolation, however, the molecular and genetic changes that occur as a result of early hormone exposures on these important cell types generally remain elusive. We aim to deepen the understanding of the effect that gonadal hormones have on the early stem/progenitor cells of the developing central nervous system—and identify underlying mechanisms behind cellular programming and maintenance of adult sex differences in the mammalian brain.

Here we present a transcriptomic approach, utilizing RNA sequencing and a global epigenetic analysis, of embryonic mouse neural stem cells (eNSCs), revealing sexual dimorphisms in gene expression at a time point prior to the onset of endogenous gonadal hormone surges, namely testosterone. In addition, we demonstrate the strong sex-specific transcriptional effects of testosterone on eNSCs, which not only equalizes numerous basal sex differences on a XX background, but serves to de-feminize and masculinize gene expression. These findings are the first to uncover basal sex differences in eNSC gene expression, and further provide a dataset of sexually dimorphic testosterone-responsive genes. Our work has also demonstrated a role of testosterone in its ability to alter epigenetic programming of eNSCs, alterations that are maintained in future daughter lineages of eNSCs. This work using our eNSC model contributes to a newly proposed mechanism of how early exposures to gonadal hormones cause cellular changes which are maintained over the life of the animal, despite considerable developmental neurogenesis and gliogenesis. These activational and persisting changes that occur as a result of hormonal exposures demonstrate the long-term effects of hormonal influence and shed light on the biological basis of sex-biased neuro-psychiatric disorders^18^.

## Results

### Generation of multipotent neural stem cells

**Figure 1:**
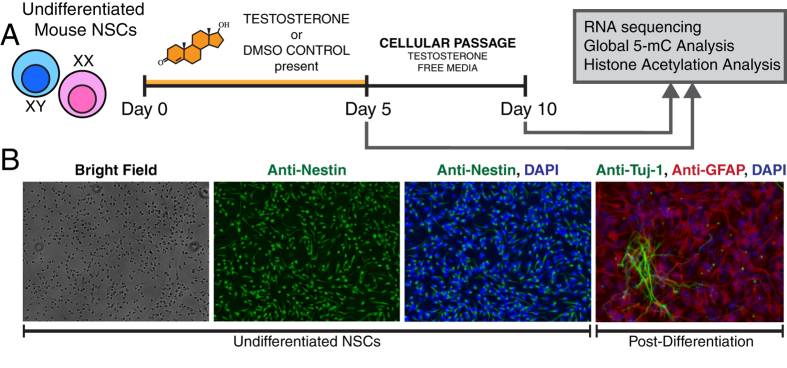
(**A**) Experimental design to explore both the activational and organizational effects of testosterone exposure on undifferentiated embryonic neural stem cells harvested from E13.5–14 male and female C57/BL6/J mice. (**B**) Immunofluorescence staining of Nestin (Green) in XX undifferentiated neural stem cells prior to differentiation, demonstrating the strong expression of this eNSC marker. Immunofluorescence staining after 21 days of differentiation, demonstrating that our eNSCs are capable of giving rise to both neurons and astrocytes as demonstrated by the neuronal marker Tuj-1/β3-tubulin (Green) and the mature astrocyte marker GFAP (Red).

To determine the transcriptomic effects of testosterone exposure on XY and XX undifferentiated eNSCs, we harvested multipotent neural stem cells from embryonic 13.5–14 C57BL6/J mice and followed the experimental timeline outlined in (Fig. 1A). After a growth period of 5 days, we stained for an accepted NSC marker, Nestin, which showed strong expression in both XX and XY cell types (Fig. 1B) demonstrating an accepted NSC staining profile. As identified by RNAseq reads, our eNSCs are also Pax6 and Sox2 positive, further confirming accepted markers of eNSCs within our experimental cell type (data not shown)^19^,^20^. In addition to staining markers of eNSCs, these cells also give rise to both mature neurons and astrocytes, as demonstrated by the GFAP and Tuj-1 staining, 21 days post-differentiation (Fig. 1B). Taken together, our immunofluorescence profile, gene expression data, and the differentiation potential, validate the multipotent characteristics of our experimental cell line.

### Male and Female Embryonic Neural Stem Cells Show Sexual Dimorphisms in Gene Expression

**Figure 2:**
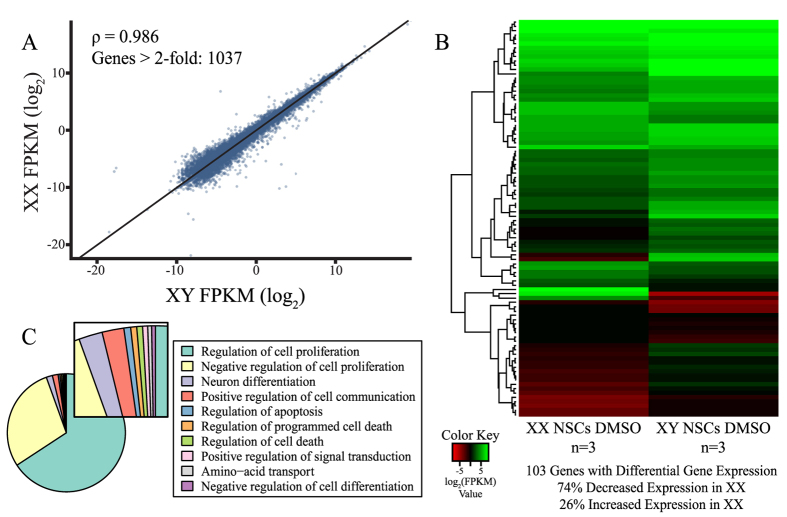
(**A**) A distribution plot representation of XX verses XY global gene expression (log2 FPKM). (**B**) Represents the 103 differentially expressed genes (FDR = 0.10) between the biological replicates of undifferentiated XX and XY embryonic neural stem cells, (**C**) The top ten most significant biological process enrichments obtained from DAVID bioinformatics analysis software for the genes identified to be dimorphic in expression between XX and XY murine eNSCs.

First, we wanted to determine if eNSCs exhibited any sexual dimorphisms with regards to gene expression, prior to androgenic hormone exposure. RNA sequencing analysis comparing XX and XY undifferentiated eNSCs found that there was a strong correlation between global gene expression (ρ = 0.986) and identified over 1000 genes that were greater than 2-fold differentially expressed between sexes (Fig. 2A). After a FDR correction of 0.10, we identified 103 transcripts that were significantly differentially expressed (Fig. 2B) (Supplemental Table 1). This effect is attributed to arise from an inherent sex chromosome effect, as these cells have not been extensively exposed to gonadal hormones prior to isolation from embryos. Of the differentially expressed transcripts in eNSCs, 74% showed a decreased expression, and 26% showed increased expression in an XX background relative to XY.

*A priori*, we expected certain genes to be differentially expressed between sexes, specifically those genes known to be expressed only in females and those present on the Y chromosome. As an internal control, we indeed found that *Kdm5d* and *Xist* were the two most differentially expressed genes between XX and XY eNSCs at baseline. Given their known sexually dimorphic expression patters^21^ further supports the validity of our data set (Supplemental Table 1). For additional confidence, we selected 12 of our differentially expressed genes to validate by a secondary approach of qPCR, which showed similar expression differences that were identified within our RNA-seq dataset (Supplemental Fig. 1). Having uncovered sexual dimorphic gene expression in embryonic neural stem cells, we queried if these 103 genes were enriched in any known biological processes. Using DAVID bioinformatics software^22^,^23^, we found that the vast majority of these differentially expressed genes were enriched in biological processes known to control cellular proliferation (Fig. 2C). Interestingly, other pathways including those involved in cellular communication and neuronal differentiation were also enriched among sexually dimorphic genes (Fig. 2C).

### Testosterone Alters Global Gene Expression in Both XX and XY eNSCs

**Figure 3:**
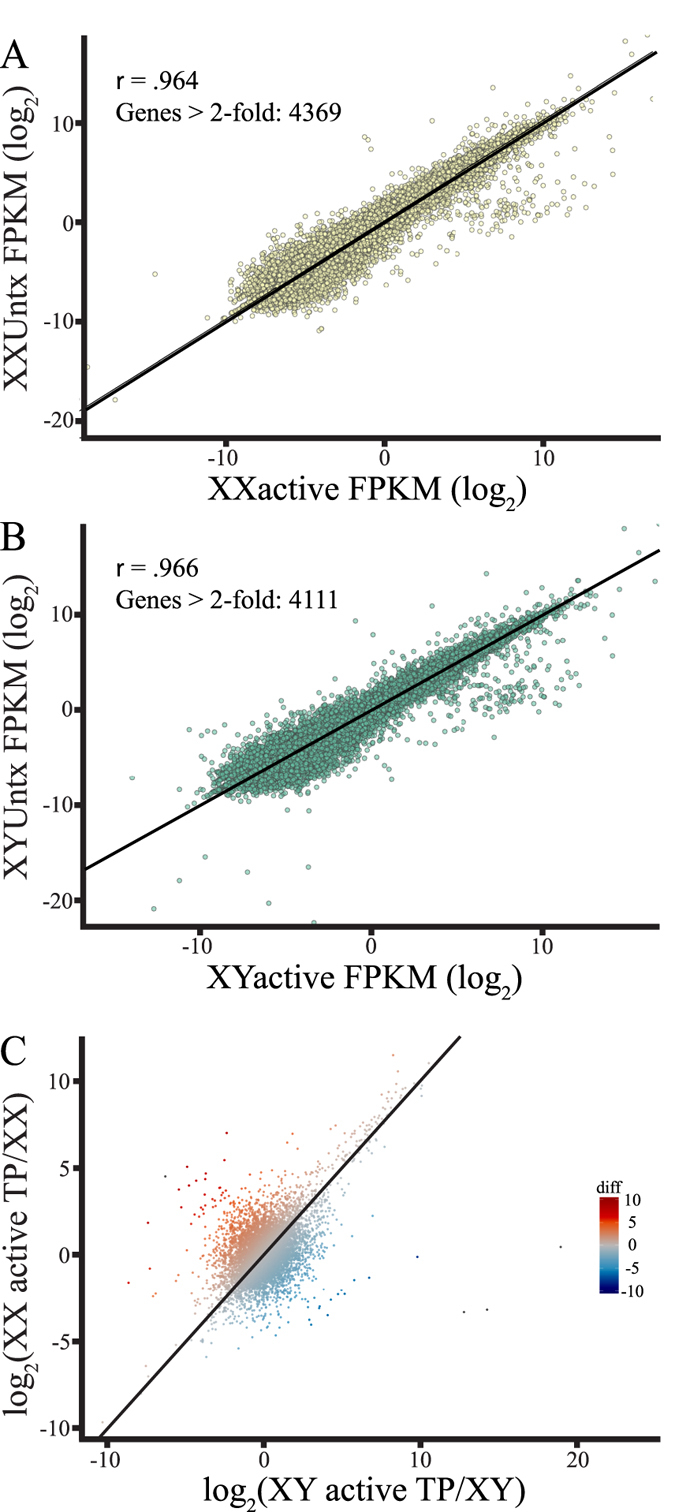
(**A,B**) A distribution of global XX or XY baseline gene expression compared (Y-axis) to XX or XY gene expression in the presence of 20 nM testosterone propionate (X-axis). (**C**) Distribution ratios comparing the effects of chromosomal sex and testosterone on global gene expression of XX in TP/baseline gene expression (Y-axis), versus global gene expression changes due to testosterone of XY in TP/baseline gene expression (X-axis).

After identifying sexually dimorphic basal gene expression differences, we next determined if testosterone had an effect on global gene expression. We treated XX and XY eNSCs with a single dose of TP to a final concentration of 20 nM for a period of 5 days^24^; RNA-seq was performed and analyzed to determine the effects of gonadal hormones on the transcriptome (Fig. 1A). We found that in both XX and XY eNSCs, testosterone had a large global effect on gene expression when compared to basal transcript levels (Fig. 3A,B). When we screened for genes that had greater than a 2-fold log_2_ change in expression as a result of testosterone exposure, we detected over 4000 such events in both sexes. We next queried the data set to determine if the effect of testosterone was the same in both sexes at the global level of transcription. To determine this, we took the log_2_ value of gene expression for both sexes in the presence of testosterone and divided that by the baseline log_2_ expression, giving us a ratio based on both the sex chromosome and testosterone effect. Once these ratios were derived and plotted, we see the effect of testosterone was markedly different between sexes (Fig. 3C). We see that the majority of genes cluster in the 1:1 regions of the plot indicating that testosterone does not affect all genes, as expected. Most interestingly however, we found that there are a significant number of gene points that are widely dispersed away from the center cluster, indicating that the effect of testosterone on altering gene expression appears to be dimorphic at the global level, depending on the sex chromosomal composition of cells that are being exposed to testosterone (Fig. 3C).

### Sex Chromosome Dependent Effects of Testosterone Exposure

**Figure 4:**
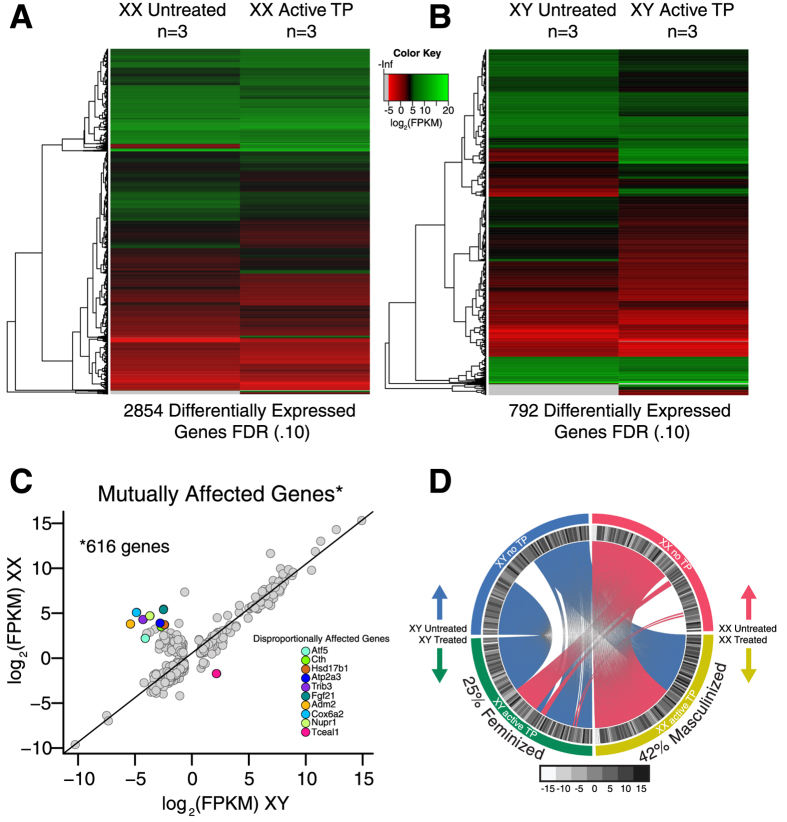
(**A**) Heat map representation of the 2854 genes that were differentially expressed on an XX background after exposure to testosterone using a FDR = 0.10, genes are clustered based on similar degrees of differential expression. (**B**) Heat map representation of the 792 differential expressed transcripts on an XY background in the presence of testosterone using a FDR = 0.10, genes are clustered based on similar degrees of differential expression. (**C**) Dot plot representation of the delta log_2_ FPKM expression values for the 616 mutually affected genes on both XX and XY backgrounds. (**D**) Circos plot representation showing the direction of masculinization or feminization of the 103 genes that were identified to be differentially expressed between XX and XY eNSCs at baseline. Genes connected by pink indicate that those genes either became or maintained a female typical expression pattern in the presence of TP. If the genes are connected by blue, this indicates that those genes either became or maintained a male-typical pattern of gene expression in the presence of TP.

Once we established that testosterone had global effects on gene expression, we determined which genes were differentially expressed in each sex utilizing a FDR = 0.10. We found that 2854 genes were differentially expressed (FDR = 0.10) in XX eNSCs post-testosterone exposure, compared to baseline expression values (Fig. 4A). Of the 2854 differentially expressed genes, half were up regulated and half were down-regulated after treatment (Supplemental Table 2). We then determined if the effect of testosterone was the same on an XY background. We found that 792 genes were differentially expressed in male eNSCs as a result of TP exposure (Fig. 4B). On this genetic background we found that 70% of genes were downregulated, and 30% of the 792 differentially expressed genes were up regulated in the presence of testosterone (Supplemental Table 3). Despite the fact that testosterone had a more robust effect on XX eNSCs than on XY, the two groups shared 616 genes that were mutually affected by TP, constituting 78% of the total genes affected in XY cells (Fig. 4C). Next, we determined if there were perhaps functional groups that were being affected within the differentially expressed genes post-TP treatment that were unique to each sex. To further analyze possible functional groupings of sexually dimorphic patterns of gene expression we subjected the top 200 most down regulated genes from each genetic background to pathway analysis using the recently available Broad Institute’s GeNets software. Each gene set was analyzed with the seven publically available algorithms (PPI, ConcensysPathDB, GeNets Metanetwork V1.0, Geo, Achillies, Blast and CLIME). Pathways identified were done so using three main criteria, 1) all associations had to be significant, 2) all associations had to have more than 2 genes in their functional groupings, 3) all associations had to be present in more than one algorithm queried. We identified two significant pathways that were unique to each sex, implicated by multiple algorithms in our post-TP exposed cells. Pathways involved in regulating potassium channels were the most downregulated in XX cells (Supplemental Fig. 2A). However, in XY cells, TP downregulated a pathway involved in internalization of an epidermal growth factor receptor, ERRB1 (Supplemental Fig. 2B). These findings demonstrate that the effect of TP exposure is distinctly different between the sexes of eNSCs, causing pathways unique to each sex to become potentially differentially regulated.

### Sex Chromosome Independent Effects of Testosterone Exposure

Having found that testosterone affected transcription in a sex chromosome-specific manner, we investigated how the 616 mutually affected genes between XX and XY were responding to TP. After plotting the delta FPKM (log_2_) expression values for both sexes pre and post-TP exposure, we found that the shared gene group responded quite similar to testosterone (Fig. 4C). The majority of the mutually affected genes responded in corresponding directions after being exposed, regardless of sex chromosome complement, as shown by the strong correlation coefficient of 0.96 (Fig. 4C). Although the majority of mutually affected genes responded in similar directions, a small but interesting group of genes responded either inversely, or with greater magnitude after hormone treatment depending on genetic background. The top ten most disproportionally affected genes that were shared between the sexes are *Atf5, Cth, Nupr1, Cox6a2, Trib3, Hsd17b1, Fgf21, Adm2, Atp2a3* and *Tceal1* (Fig. 4C). Having identified enriched functional groups that were uniquely downregulated in each sex (Supplemental Fig. 2A,B), we next determined pathways which were up-regulated as a result of TP exposure irrespective of sex chromosome complement. We queried if the 200 most up regulated genes in the mutually affected set were enriched in any known biological functional categories^22^,^23^. We found a strong enrichment in pathways representing nucleosome organization/assembly and general DNA architecture which were comprised of three communities of gene groups that were significantly upregulated in both XX and XY embryonic neural stem cells after hormone treatment (Supplemental Fig. 2C). These enriched pathways that are altered in eNCSs from exposures to androgenic hormones are important for both DNA compaction in addition to how the genetic code is regulated, transcribed and translated.

### Testosterone has Both Masculinizing and Feminizing Effects in eNSCs

The effects of testosterone on the brain are often characterized in terms of how masculinized a trait becomes over time, such as the case in the hormonal organization theory. We investigated if the basal sex differences that were previously identified (Supplemental Table 1), had become masculinized (more similar to XY transcript reads) or feminized (more similar to XX transcript reads) in gene expression after TP treatment. We found that 43 out of the 103 original sex differences post-TP exposure on a XX background were at least equal to the FPKM expression values for the same gene on a XY background prior to TP treatment, indicating full masculinization of such gene expression (Fig. 4D, Supplemental Table 1). Unexpectedly, we also identified 26 genes on a XY background that actually became completely feminized as a result of exposing such cells to TP (Fig. 4D, Supplemental Table 1). These feminizing and masculinizing events as a result of exposing embryonic neural stem cells to a male sex hormone are perhaps identifying key genes responsible for establishing typical male gene expression within the cells of the developing central nervous system. In addition to identifying key genes involved in typical-male brain development, the findings also shed light on early transcriptional events that may be occurring in hyper-masculinized female development, as seen in cases of XX congenital adrenal hyperplasia (CAH). These findings demonstrate the ability of gonadal hormones to modulate effects of sex chromosome composition, resulting in diminished XX and XY basal gene expression differences, making such expression more similar in nature.

### Long-term Effects of Testosterone Exposure on the Transcriptome of eNSCs

After we identified that testosterone was able to alter gene expressing in both sexes of eNSCs we next determined if these gene expression differences were maintained in the daughter cells of the TP exposed eNSCs. We found that once the cells were passaged and allowed to grow for an additional 5 day period prior to RNA-sequencing in the absence of testosterone (Fig. 1A), no differential gene expression was maintained using an FDR = 0.10. These data show that while testosterone does have a strong activational effect with regard to altering gene expression; these effects are not maintained within in the daughter cell population in the absence of testosterone, indicating that these transcriptional effects occur only while the hormone is present (data not shown).

### Testosterone Triggers a DNA De-methylation Event that is Maintained in Daughter Cell Linages of eNSCs

**Figure 5:**
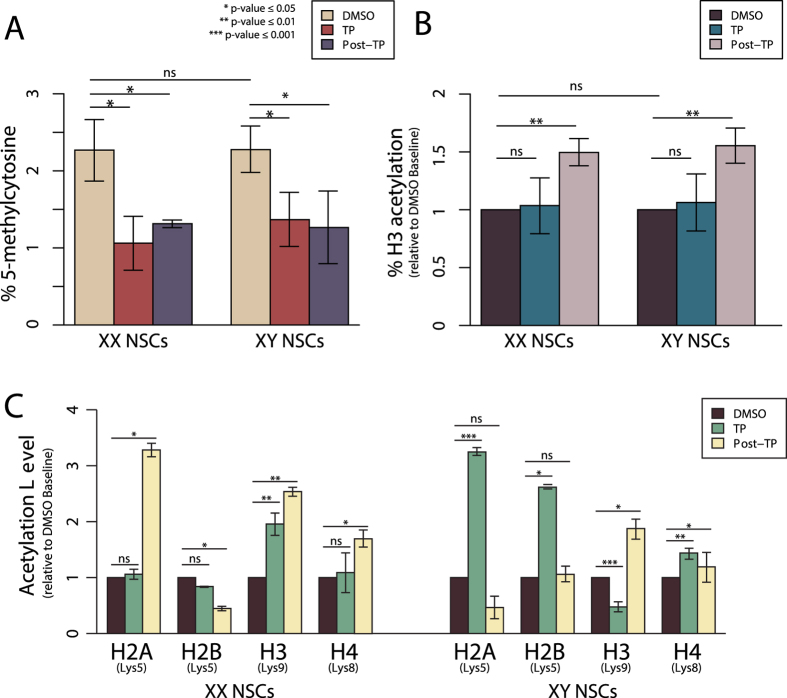
(**A**) Global 5-methylcytosine analysis of XX and XY eNSCs pre TP exposure, during TP exposure and within the daughter cells post-TP exposure. (**B**) Global Histone H3 acetylation analysis of XX and XY eNSCs pre-TP exposure, during TP exposure and within the daughter cells post-TP exposure. (**C**) Bar plot representation of residue-specific western blot analysis assessing acetylation abundance during active exposure to TP and within the daughter cells post-TP exposure relative to their corresponding control (DMSO) populations which have been set to a value of 1.

Since epigenetic modifications are beginning to be linked to the organization of the mammalian brain, we wanted to investigate if in addition to altering gene expression, was testosterone able to alter global 5-methylcytosine abundance within eNSCs. We first assessed if there were baseline differences in global DNA methylation abundance between XX and XY eNSCs. Using an ELISA based approach, we determined that the global DNA methylation percentage in XX cells was 2.27% and 2.28% in XY eNSCs (Fig. 5A). These findings indicate that unlike gene expression, baseline global DNA methylation between the sexes is not sexually dimorphic in global percentage levels. After exposing the cells to testosterone for a 5-day period (Fig. 1A), we found that testosterone has a strong effect in reducing the levels of DNA methylation by roughly 50% in both XX and XY cells. We found that during TP exposure, global DNA methylation dropped to 1.06% (p = < 0.05) on a XX background and 1.37% (p = < 0.05) on a XY background (Fig. 5A). Most interestingly however, this reduction of DNA methylation was maintained within the daughter cell linages of TP exposed neural stem cells. We identified that the XX daughter cells from the previously exposed parental cells had a global DNA methylation percentage of 1.31% (p < 0.05) and the XY daughter cells had 1.26% (p < 0.05) (Fig. 5A). These findings indicate that testosterone actively reduces DNA methylation levels in embryonic neural stem cells, but most importantly demonstrates a long-term effect on altering the DNA epigenome in subsequent cellular linages after NSC replication from TP exposed cells.

### Testosterone affects the Global Acetylation Pattern of Histone H3 in Daughter Linages of TP Exposed NSCs

After demonstrating that testosterone was capable of affecting global DNA methylation patterns, we next sought to determine if TP was capable of altering other epigenetic regulatory modifications in eNSCs. Using a similar ELISA-based approach, we determined if testosterone affected the global percentage of acetylation on histone H3. We found again that there were no significant differences in global H3 acetylation level between XX and XY embryonic neural stem cells at baseline (Fig. 5B). Unlike our methylation results, we saw no significant changes in global H3 acetylation levels in either sex during active TP exposure, with XX cells being at 103% (p = 0.83) and XY cells being at 106% (p = 0.72) of baseline (Fig. 5B). Surprisingly, we observe a dramatic increase in global H3 acetylation in both XX and XY daughter cells derived from the actively exposed parental linages. Specifically, Post-TP XX eNSCs show a 50% acetylation increase (p < 0.01) compared to baseline and Post-TP XY eNSCs were identified as have a 55% increase (p < 0.01) in acetylation compared to baseline (Fig. 5B). These data demonstrate there are late-emerging global hyper-acetylation events occurring within this progenitor cell population as a result of testosterone exposure.

### Testosterone Causes Sexually Dimorphic Residue-Specific Alterations of Histone Acetylation levels

Once it was established that testosterone was capable of altering global acetylation in eNSCs, a more site-specific analysis of lysine residue acetylation was conducted to determine if specific residues in both XX and XY eNSCs were differentially responsive to TP exposure. Using a western blot approach, we saw no significant changes in Lys9 of histone H2A, Lys5 of histone H2B, and Lys8 of histone H4 as a result of active TP-treatment in a XX eNSC background (Fig. 5C) (Supplemental Fig. 3A). In contrast, Lys9 on histone H3 was elevated 2-fold as a result of direct TP exposure (p < 0.01) (Fig. 5C). Furthermore, analysis of the post-treatment XX NSCs demonstrated significant changes in acetylation levels at specific residues in the daughter cells of previously exposed eNSCs. Specifically, we report a 3-fold increase in Lys5 of H2A acetylation (p < 0.05), over a 1-fold decrease of acetylation on Lys5 of H2B (p < 0.05), maintained hyper-acetylation in Lys9 of H3 (p < 0.01) and a 1.5-fold increase in Lys8 of H4 (p < 0.05), all relative to the corresponding acetylation levels in post-treated control samples (Fig. 5C) (Supplemental Fig. 3B).

Unlike in XX cells, XY eNSCs showed significant changes in residue-specific acetylation on all core histones during active hormone treatment. Compared to baseline, we saw a 3.25-fold increase in acetylation on Lys5 of H2A (p < 0.001), a 2.5-fold increase in Lys5 of H2B (p < 0.05), a 50% reduction in acetylation levels on Lys9 of H3 (p < 0.001) and a 1.5-fold increase in Lys8 of H4 (p < 0.01) (Fig.5C) (Supplemental Fig. 3C). Additionally, in the post-treatment daughter cells we also observed different outcomes than observed on a XX background. Again, compared to control post-treated cells, we see that acetylation levels of Lys5 of H2B revert back to baseline, Lys9 of H3 shows a 1.9-fold increase (p < 0.05) and Lys8 of H4 shows a 1.2-fold increase (p < 0.05). On Lys5 of H2A there is a ~50% reduction in acetylation levels, however these findings did not reach statistical significance (p = 0.1) (Fig. 5C) (Supplemental Fig. 3D). Taken together, we see that active exposure to testosterone has both residue-specific as well as sex-specific effects on the acetylation abundance of core histone protein residues. Additionally, the effects of testosterone are not only transient, but can also affect acetylation levels in daughter cells from previously exposed eNSCs of both sexes.

## Methods

### Animal Care and Neural Stem Cell isolation

C57BL/6/J XX and XY mice were purchased from Jackson Laboratories (Bar Harbor, ME, USA) and housed at the UCLA Animal Care Facility. Animals were maintained at 20 °C with a 12-h light/12-h dark cycle, provided *ad libitum* with food and water. The study was approved by the University of California, Los Angeles (UCLA) Committee on Animal Research and was performed in accordance with the recommendations in the Guide for the Care and Use of Laboratory Animals of the National Institutes of Health. At eight weeks of age three dams and three males were mated separately to generate the biological triplicate basis, and the gestation was timed for 13.5 days. After the appropriate gestation time, the pregnant dams were humanely sacrificed, and immediately prepped for embryo extraction. Embryos were separated, followed by the removal of the brain which was then placed separately into dishes containing cold PBS/2% glucose. The ganglionic eminences from both hemispheres were removed and placed into 1.7 ml Eppendorf tubes supplemented with cold PBS/2% glucose. This procedure was repeated for every embryo in each of the three dams following standard technical protocols^25^. Following isolation of the ganglionic eminence regions, the CNS tissue in each tube was triturated using a P-200 until no tissue clumps were visible prior to being passed through a 40 μM cell strainer. After straining, all cells from each brain region were plated into 6-well culture dishes (Falcon) and suspended in Complete Embryonic Neurocult^TM^ Proliferation Media (STEMCELL Technologies) supplemented with 20 ng/ml rhEGF (STEMCELL Technologies). The cells were allowed to incubate at 37 °C/5% CO_2_ for a period of 3–5 days until sphere formation began. Once cells had formed spheres they were sexed, then dissociated using ACCUTASE^TM^ (STEMCELL Technologies) and passaged for immediate experimentation or cryopreserved for downstream investigation. Sexing primers were as follows: mSRY:5′CATTTATGGTGTGGTCCCGTG3′, 5′CTCTGTGACACTTTAGCCCTC3′, GAPDH 5′TGACCTCAACTACATGGT3′, 5′CAGTGGATGCAGGGATGA3′.

### Testosterone Treatments

3 independent XX and XY primary mouse neural stem cell lines were brought to single cell suspension using ACCUTASE^TM^ (STEMCELL Technologies), and 1.0 × 10^6^ cells were seeded into a 10 cm non-adherent culture dish. The cells were suspended in Complete Embryonic Neurocult^TM^ Proliferation Media (STEMCELL Technologies) supplemented with 20 ng/ml rhEGF (STEMCELL Technologies) and a single addition of TP yielding a final concentration of 20 nM Testosterone Propionate (Sigma) on day 1, or the corresponding volume of DMSO vehicle (final concentration of 1.3 × 10^−4^%)(Sigma). The cells were allowed to replicate for a period of 5 days in each media type prior to being prepped for RNA extraction or passaged. After a 5 day growth period a subset of cells that were not used for RNA extractions were brought to a single cell suspension and 5 × 10^5^ cells were seeded onto 10 cm non-adherent culture dishes. These cells were suspended in Complete Embryonic Neurocult^TM^ Proliferation Media (STEMCELL Technologies) supplemented with 20 ng/ml rhEGF (STEMCELL Technologies), without the addition of TP or DMSO. After an additional 5 days these cells were prepared for RNA sequencing.

### RNA extraction

After 5 days of growth the NSCs in TP and DMSO were pelleted and washed 1x in PBS and then re-pelleted. The washed pellet was re-suspended in Buffer RLT-Plus (Qiagen), and then RNA was extracted following the standard protocol for the RNeasy® Plus Mini Kit (Qiagen). Post extraction the RNA was subjected to DNase treatment for 30 min using the TURBO DNA-free^TM^ (Ambion). RNA integrity was assessed using the Agilent RNA 6000 Nano Kit (Agilent Technologies) following standard protocols at the UCLA Genotyping and Sequencing Core. All RNA samples that were used in these studies had RIN values greater than 8.0.

### Library Preparation and RNA Sequencing and Expression Analysis

1 ug of total RNA from each sample was submitted to the UCLA Neuroscience and Genomics core (UNGC) for cDNA library preparation. Unpooled cDNA sequencing libraries were prepared using TruSeq Stranded Total RNA with Ribo-Zero (Illumina) following standard protocols. 8, 4 and 6 libraries were multiplexed and sequenced on an Illumina HiSeq 2500 next generation sequencing machine on three different runs. Sequencing reads were aligned using TopHat (v2.0.8b)^26^ to the mouse genome (mm9) and Ensembl Mus Musculus GTF file (Mus musculus NCBI37). Each sample had an average of 36,067,841, 35,519,612 and 55,760,848 unique reads mapped with a frequency greater than 80.11%, 89.82% and 88.73% being successfully aligned to the reference genome. Cufflinks (v2.1.1) was used to find gene expression per sample and Cuffdiff from Cufflinks was used to find differential expression. A false discovery rate of 0.10 was implemented as the statistical analysis used to determine significance of differential gene expression depending on whether the uncorrected p value was greater than the FDR after Benjamini-Hochberg correction for multiple-testing. Each comparison included 6 samples at two different conditions. Conditions were equally distributed among the three runs.

### Quantitative-PCR Validation

1 μg of total RNA that had been previously extracted for RNA-sequencing was converted to cDNA using the High Capacity RNA-to-cDNA Kit (Applied Biosystems) following manufactures protocol. Post conversion, the final volume of cDNA was diluted 1:4 using nuclease-free water and pooled. Quantitative-PCR reactions were prepared using the PowerUp™ SYBR™ Green master mix (Applied Biosystems) following manufactures protocol, with the addition of 3 ul of diluted cDNA and the appropriate forward and reverse primers for the gene of interest. All qPCR reactions were ran and analyzed on a Bio-Rad CFX-Connect Real-Time System. Cq values for control sample and testosterone exposed samples were normalized to Cq values for the housekeeping gene Beta-2-Microglobulin (B2M). Fold changes were determined by subtracting the experimental testosterone groups Cq from the Cq of the corresponding gene in the control group. Each analysis was run in technical quadruplicate for both the control and testosterone samples followed by a Student’s t-test to determine statistical significance of fold-change for each tested gene.

### Pathway analysis

The 103 genes that were found to be sexually dimorphic prior to hormone exposure were all subjected to pathway enrichment using DAVID bioinformatics software. The top 10 most statistically significant enrichments were tabulated using the biological process software feature. To determine unique pathways that were downregulated in each sex post-hormone treatment, algorithms from the Broad insitutues GeNETS software (PPI, ConcensysPathDB, GeNets Metanetwork V1.0, Geo, Achillies, Blast and CLIME) were implemented for the top 200 most down regulated genes in each sex. For the mutually upregulated pathways both DAVID bioinformatics software and algorithms from the Broad institute’s GeNETS software (PPI, ConcensysPathDB, GeNets Metanetwork V1.0, Geo, Achillies, Blast and CLIME) specifically the Metanetwork V1.0 were used in that analysis.

### Global DNA 5-mC Quantification Assay

DNA methylation was assessed using the MethylFlash Methylated DNA Quantification Kit (Epigentek) following manufactures protocols. Assays were conducted using 100 ng of DNA that was isolated using the gDNA^TM^ MiniPrep kit (Zymo Research) following manufactures protocols. DNA was quantified prior to experimentation using a Qubit® fluorometer with the Qubit® dsDNA BR assay reagents. Samples were ran as three biological replicates in technical triplicate and final absorbance readings were subjected to a Welch two subject t-test to establish statistical significance for each comparison.

### Global H3 Acetylation Assay

Global H3 acetylation was determined using the EpiQuik™ Global Histone H3 Acetylation Assay Kit (Epigentek) following manufactures protocols. Protein concentration of the nuclear extracts was quantified using a BCA assay, and 1.5 μg of total protein was used from each experimental group. Samples were run using three biological replicates in technical triplicate and final absorbance readings were subjected to a Welch two subject t-test to establish statistical significance for each comparison. Acetylation levels were displayed as a ratio compared to the corresponding control H3 acetylation abundance.

### NSC preparation for residue-specific acetylation assay

3 independent XX and XY primary mouse neural stem cell lines were brought to single cell suspension using ACCUTASE^TM^ (STEMCELL Technologies), and 1.0 × 10^6^ cells were seeded into a 10 cm non-adherent culture dish. The cells were suspended in Complete Embryonic Neurocult^TM^ Proliferation Media (STEMCELL Technologies) supplemented with 20 ng/ml rhEGF (STEMCELL Technologies) and with the addition of a final concentration of 20 nM Testosterone Propionate (Sigma), or the corresponding volume of DMSO vehicle (Sigma). The cells were allowed to replicate for a period of 5 days in each media type prior to being prepped histone extraction. After a 5 day growth period a subset of cells that were not used for histone extractions were brought to a single cell suspension and 5 × 10^5^ cells were seeded onto 10 cm non-adherent culture dishes. These cells were suspended in Complete Embryonic Neurocult^TM^ Proliferation Media (STEMCELL Technologies) supplemented with 20 ng/ml rhEGF (STEMCELL Technologies) but without the addition of TP or DMSO. After an additional 5 days of growth these cells were prepared for histone extraction.

### Histone extraction for residue-specific acetylation assay

2 × 10^6^ cells from each NSC sample was collected and centrifuged at 1100 RMP for 10 min. The cellular pellets were washed 2X using DBPS and re-pelleted at 1100 RPM. The final washed pellets were re-suspended in 750 μl lysis buffer (10 mM HEPES (pH 7.9), 1.5 mM MgCl_2_, 10 mM KCL, 0.34 M sucrose, 10% glycerol, 1 mM DTT, 1X protease inhibitor and 0.1% Triton-X). Cells were allowed to lyse on ice for 10 min. Samples were then centrifuged at 4,500RPM for 5 min at 4 °C. Supernatant was removed and the pelleted nuclei were re-suspended in an additional 750 μl of lysis buffer. Nuclei pellets were centrifuged at 4,500RPM for 5 min at 4 °C followed by the aspiration of supernatant. Pellets were re-suspended and triturated in 50 μl of histone extraction buffer (10% glycerol, 1.2% sulfuric acid, and 0.2% 2-mercaptoethanol). Samples were incubated on ice for 10 min then centrifuged at 13,000RPM for 10 min at 4 °C. Supernatants from the samples were collected and 12.5 μl of tricholoroacetic acid (TCA) was added. Samples were vortexed and centrifuged at 13,000RPM for 10 min at 4 °C. Supernatant was removed and mixed with 1 mL of cold 100% ethanol then chilled at −80 °C for 10 min followed by centrifugation at 13,000 RPM. The ethanol was aspirated then the samples were allowed to air-dry overnight. The dried pellets were stored at −80 °C for downstream experimentation.

### Histone quantification and normalization for residue-specific acetylation assay

Histone pellets were re-suspended in 100 μl of ddH_2_O. 30 μl of suspended histones were quantified using the Pierce™ BCA Protein Assay (Thermo Fischer Scientific) following manufactures protocols. Standard curves for protein quantification were established using albumin standards (Thermo Scientific). Quantifications of samples were run in triplicate to establish a preliminary protein concentration. The 70 μl of remaining protein sample was mixed with 4X LDS protein loading buffer and boiled for 10 min in preparation for downstream PAGE analysis. Using the preliminary protein concentrations from the BCA assay, 0.9 μg of protein from each sample group comparison (i.e XX Controls verses XX in TP) was loaded in duplicate onto a 15% SDS polyacrylamide gel and ran at 60 V for 15 min then 150 V until appropriate protein separation occurred (~60 min) in 1X Tris-Glycine buffer. The gel was washed in ddH_2_O 3X for 5 min each, then total protein was stained using SimplyBlue™ SafeStain (Invitrogen) for 1 hr. The gel was de-stained overnight in ddH_2_O and then imaged using a ChemiDoc MP Imaging System (Bio-Rad). Total protein densitometry for each lane was determined using ImageJ software (NIH) and statistical analysis of the pairwise groupings on each gel was determined using a Welch two sample t-test. Protein concentration was adjusted until the groups were statistically insignificant from one another, prior to western blot analysis used to determine acetylation levels.

### Western blot analysis for the residue-specific acetylation assay

0.9 μg of sample was separated on a 15% SDS polyacrylamide gel and ran at 60 V for 15 min then 150 V until proper protein separation had occurred (~60 min), in 1X Tris-Glycine buffer. The gel was then transferred onto a 0.45 μM PVDF membrane using a Bolt® Mini Gel Tank Transfer Device (Thermo Fisher Scientific) at 30 V for 3 hours in 1X Tris-Glycine buffer with the addition of 20% methanol. Membranes were blocked overnight at 4 °C in 1X PBS with the addition of 5% NFDM. Post blocking, membranes were incubated overnight at 4 °C with corresponding primary antibodies at a 1:500 dilution from the Acetyl-Histone Antibody Sampler Kit (Cell Signaling Technology). Membranes were then washed 3 × 10 min in PBST before being incubated 1 hr in appropriate secondary antibody at a 1:15,000 concentration. Membranes were then washed 3 × 10 min in PBST prior to imaging. Pierce™ ECL Western Blotting Substrate was used at a 50:50 concentration to activate the HRP-conjugated secondary. Luminescence was measured using a ChemiDoc MP imaging system (Bio-Rad). Samples were analyzed using three biological replicates ran in technical duplicate. Band intensities were determined using ImageJ software (NIH) and sample values were subjected to a Welch two-sample t test to determine significance between experimental groups. Data is expressed as ratios of experimental samples compared to corresponding control samples.

## Discussion

Determining the mechanism by which gonadal hormones shape the developing brain and organize the central nervous system remains to be fully elucidated. Using an *in vitro* system, we sought to better understand the effect of testosterone on the transcriptome and epigenome of the cells responsible for generating the central nervous system, eNSCs. We initially determined that gene expression between XX and XY eNSCs was sexually dimorphic, and 103 transcripts were uncovered as displaying differential expression (FDR = 0.10). These basal sex differences appear to be enriched in pathways most involved in both positively and negatively regulating cellular proliferation. This is corroborated by several recent studies which demonstrated that gonadal hormones can affect NSC proliferation^14^,^27^. Therefore, we provide evidence here that basal gene expression differences between XX and XY cells may contribute to differences in proliferation of NSCs in the absence of testosterone.

The gene expression differences in eNSCs identified here are the first documented cases of sexual dimorphism in gene expression between the sexes at this early stage within cells that comprise early CNS development. Unlike other studies that have focused on sex differences in the brain caused by genetic makeup^6^,^21^,^28^,^29^, our approach used a very specific cell type as opposed to gross brain tissue, as the latter may lack the sensitivity to observe nuances in gene expression, which may be masked by heterogeneous cell populations. This limitation has been observed when looking at DNA methylation in the brain, as it was found that global profiles are striking different between individual cells and that of heterogeneous cell populations within whole brain tissue^30^. These findings also allude to the possibility that sex differences are fluid, and can be variable over developmental time and specific to regions of interest.

After determining that eNSCs show sexual dimorphism in gene expression, we wanted to determine if these cells also responded differently to the exposure of testosterone. Again, using a complete transcriptomic approach of RNA-sequencing, we determined that testosterone has differential effects on embryonic neural stem cells depending on sex chromosome composition. We found that upon exposure to testosterone XX NSCs differently expressed over 2800 transcripts, whereas in XY cells nearly 800 transcripts became differentially regulated (FDR = 0.10). The effects of gonadal hormones on these cell types are just beginning to be uncovered, as such; this is the first documentation of global gene changes in eNSCs as a result of testosterone exposure during a developmentally relevant time in gestation^31^. It appears that TP on a XX background uniquely downregulates pathways involved in potassium channel signaling, but, on an XY background pathways involved in ERRB1 internalization are uniquely down-regulated (Supplemental Fig. 2). Some of the effects of TP are also mutual between sexes. We identified 616 transcripts that were mutually upregulated upon active testosterone exposure. Interestingly these upregulate transcripts were most enriched in pathways that were involved in gene transcription, regulation, and genomic architecture in both XX and XY eNSCs (Supplemental Fig. 2). This finding adds to the growing body of literature demonstrating that TP can indeed cause increased proliferation events in this cell type. Since there are variable outcomes in gene expression after TP exposure depending on sex chromosome complement, inherent epigenetic programming may be involved which has set the stage for differential response to such hormones in each sex.

By exposing XX eNSCs to TP, we were able to eliminate and masculinize 42% of the basal sex differences that were identified prior to hormone treatment. These findings identify that TP can elicit masculinizing events in this cell type, again adding an unknown component of androgen function within the cells abundant during early stages of brain development. The masculinizing effects of testosterone on the brain when administered to a female rodent during development, or at birth have been extensively documented^2^,^32^ however, the mechanisms of how such effects are maintained into adulthood still remain unclear. We also found that 25% of basal sex differences were completely feminized on a XY background post-TP exposure, an unexpected finding, as the feminizing effects of testosterone are not often researched. This finding is of great interest as it opens the possibilities for a sex-specific regulation of dimorphic genes in embryonic neural stem cells as a result of hormone exposures at various time points in development. For humans *in utero* testosterone exposure can have both feminizing and masculinizing effects depending on sex, as seen in our neural stem cell model. Research has shown that despite high/above average levels of testosterone during development, XY males with congenital adrenal hyperplasia (CAH) show reduced male-typical personality characteristics^33^ and reduced visuospatial performances^34^,^35^ a male dominated strength. Females with CAH typically display the reverse in these personality^33^ and cognitive^34^,^35^ abilities, shifting them towards a more male-typical pattern. These human observations raise the possibility that the timing and dose of TP exposure is critical for establishing male-typical neural outcomes in development.

As our goal was to better understand hormonal organization, we next investigated if gene expression in the daughter cells of the parentally exposed eNSCs maintained long-term gene expression changes. We determined that testosterone was unable to cause maintained transcriptional differences in the daughter cell linages, in the absence of testosterone. While the active effects of testosterone on the transcriptome are robust in both sexes, it appears that these differences revert back to near baseline if TP is removed from downstream cellular lineages. This finding prompted us to next investigate how testosterone altered the epigenome in eNSCs, as differences have been observed in DNA methylation and histone modifications in sexually dimorphic regions of the whole brain^8^,^10^,^36^.

We found that at baseline, XX and XY eNSCs do not appear to be sexually dimorphic in overall global 5-methylcytosine abundance. However, once exposed to testosterone both XX and XY cells show a dramatic decrease in global DNA methylation. This finding is also in line with recent literature showing that estradiol, a possible derivate of testosterone, if aromatized, globally causes hypomethlyation events in specific regions of the brain^8^. These events have been attributed to a reduction in DNMT activity rather than an active de-methylation event. Most interesting however, we uncovered that this global hypo-methylation is maintained in daughter cells of TP exposed eNSCs, showing long-term and possibly permanent remodeling of the epigenome of embryonic neural stem cells. In addition to affecting DNA methylation, testosterone exposure was also capable of eliciting sexually dimorphic changes in histone acetylation abundance. We identified both global and residue-specific alterations that occurred during active TP exposure. Again, what was most striking were the alterations in histone acetylation continued to be affected in the daughter cells of the TP exposed parental cellular linage, showing another long-term epigenetic effect of TP.

The role of testosterone in masculinizing the rodent brain is traditionally thought to be less significant when compared to estradiol, the primary masculinizing agent of the rodent brain. But strong evidence has shown that mice lacking a functional androgen receptor (Tfm) are not completely masculinized in numerous adult behaviors, despite having functional estrogen receptors^37^ Tfm mice display a de-masculinized pattern of copulation as compared to their wild type counterparts^38^ which initially established the role that androgen receptor was required for complete masculinization of the rodent brain. Due to the fact that the cell type in this particular study is α/β estrogen receptor negative, as well as negative for P450 aromatase, it seems likely that the dramatic transcriptional and epigenetic changes that ensues within eNSCs post-TP exposure is mediated by action on the androgen receptor (AR), as this is the only transcriptionally detectable hormone receptor relevant to the study. However, additional experimentation is necessary to fully determine if the effects of TP observed in eNSCs are solely due to action on AR and not attributed to other elusive mechanisms.

**Figure 6:**
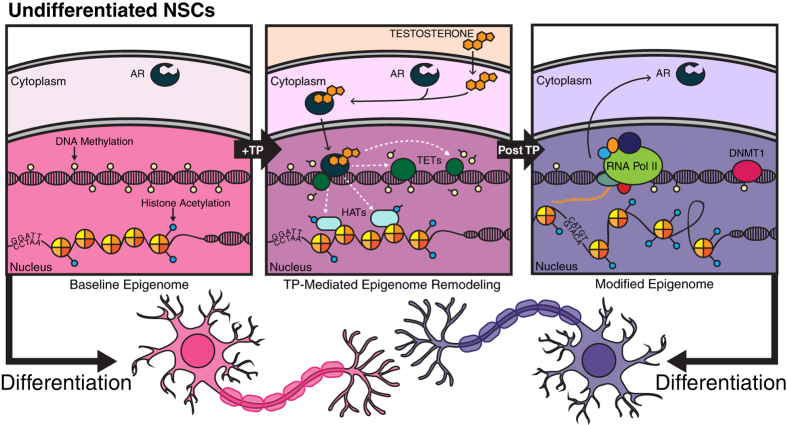
A proposed mechanism of early hormonal organization by which testosterone alters both the transcriptome and epigenome of neural stem cells, setting the stage for downstream effects in their differentiated progeny. Testosterone exposure results in large transcriptional effects that can alter epigenetic machinery and general DNA architecture. The exposure of T likely causes an active hypo-methylation event and a remodeling of histone tale acetylation abundance. After cellular division this altered epigenetic programming is maintained thereby generating population of eNSCs which harbor re-programmed cellular memory and nuclear architecture. Upon differentiation it is likely that these epigenetic and transcriptional alterations effect both astrocytes and neurons, and “primes” them for complete masculinization or feminization during various time points of critical development.

These findings in eNSCs raise a completely new role for testosterone action within the early cells of the CNS, and have led to the proposal of a mechanism by which hormonal organization is able to be maintained through adulthood. Testosterone is capable of eliciting large transcriptional changes in eNSCs; thereby affect the epigenetic machinery of the cell. By doing so, TP possibly causes an active de-methylation event via the activation of TET proteins, as our RNA-seq data identified over a 1 fold increase in TET2 mRNA expression in the presence of testosterone (Supplemental Tables 2 and 3). In addition to DNA methylation, histone residues are also modified either by increasing or decreasing acetylation levels at specific sites. Collectively, these epigenetic changes are maintained within the daughter cells, thereby creating new populations of eNSCs that now contain a modified epigenome. Upon differentiation large epigenetic events occur, and it is not unfathomable that these TP induced modifications of eNSCs will ultimately affect the downstream epigenomes of both the differentiating neurons and astrocytes. These modified differentiated cells may now be “epigenetically primed” and once exposed to estradiol at later time points in development, may become fully masculinized and enable variable adult behavioral phenotypes (Fig. 6). This possible mechanism could explain how despite significant neurogenesis over development the effects of *in utero* testosterone exposure are maintained and able to alter adult behavior. Since all CNS cells arise from eNSCs, if these populations have been re-programmed via gonadal hormone exposure, during self-renewal and differentiation the epigenetic changes that occurred early in development are maintained over the life of the mammal.

Our proposed mechanism of TP action on the early CNS will require additional downstream work, however, the evidence that we have uncovered alludes to a strong possibility that genetic/epigenetic modifications in eNSCs could permanently affect downstream cellular lineages on many different measures. Additionally, the elucidation of these basal sex differences and the sex-specific effects of testosterone on both the transcriptome and epigenome will contribute greatly to a new focus of study within the fields of neuroendocrinology and behavioral neuroscience.

## Additional Information

**How to cite this article**: Bramble, M. S. *et al*. Sex-Specific Effects of Testosterone on the Sexually Dimorphic Transcriptome and Epigenome of Embryonic Neural Stem/Progenitor Cells. *Sci. Rep.* **6**, 36916; doi: 10.1038/srep36916 (2016).

**Publisher’s note:** Springer Nature remains neutral with regard to jurisdictional claims in published maps and institutional affiliations.

## Supplementary Material

Supplementary Information

Supplementary Information

Supplementary Information
