# Supplementary material for: Sex-Specific Effects of Testosterone on the Sexually Dimorphic Transcriptome and Epigenome of Embryonic Neural Stem/Progenitor Cells
Source: Sci Rep. 2016 Nov 15;6:36916. doi: 10.1038/srep36916 (PMC5109279; doi:10.1038/srep36916)
Supplement: Supplementary Information [file srep36916-s1.pdf]

Sex-Specific Effects of Testosterone on the Sexually Dimorphic Transcriptome and Epigenome of Embryonic Neural Stem/Progenitor Cells

Matthew S. Bramble[1], Lara Roach[1], Allen Lipson[1], Neerja Vashist[1], Ascia Eskin[1], Tuck Ngun[1], Jason E. Gosschalk[1], Steven Klein[1], Hayk Barseghyan[1], Valerie A. Arboleda[1,2] and Eric Vilain[1]

[1]Department of Human Genetics at the David Geffen School of Medicine, University of California Los Angeles, Los Angeles, CA. 90095
[2]Department of Pathology and Laboratory Medicine, at the David Geffen School of Medicine, University of California Los Angeles, Los Angeles, CA. 90095

(Supplemental Figure File)

Supplemental Table 1: Baseline Sex Differences and Post TP FPKM log_2 Expression Values

Gene	XX Baseline	XY Baseline	XX+TP	XY+TP
2700007P21Rik	4.34	5.79	6.57	4.82 ¥
1700008F21Rik	-3.60	0.50	-0.38	-2.82
AC103359.1	-Inf	-0.83	0.71	-1.55 ¥
Abcc4	2.23	4.14	3.30	2.06 §
Adm	4.32	5.38	5.48	4.45 ¥
Adm2	-1.44	3.29	2.35	-2.13 §
Adora2a	-1.83	0.44	-0.82	-2.49 §
Afap1l2	1.75	3.40	4.25	2.96 ¥
Agap2	4.34	3.43	2.92	3.82 ¥
Agt	3.40	2.08	3.86	3.07
AI464131	3.36	2.32	3.03	2.66
Anxa6	3.97	4.93	4.04	4.61
ApoE	9.30	8.18	9.52	9.89 §
Atf4	7.06	8.02	8.60	7.42 ¥
Atf5	4.88	8.02	7.11	3.86 §
Atp2a3	-3.43	-0.19	0.45	-3.01 ¥
Atp2b4	2.01	0.36	0.74	1.23
Barhl2	-0.47	1.27	-0.01	1.17
Bdnf	-1.27	0.41	1.19	-0.37 ¥
Cadps	0.19	2.26	1.19	1.00
Car3	6.80	5.65	5.69	6.42
Cd24a	5.65	6.57	6.40	6.00
Cd36	2.49	0.52	1.39	0.71
Cebpg	3.78	5.26	5.80	4.21 ¥
Chac1	2.22	4.57	6.09	3.39 ¥
Col28a1	-0.17	1.93	1.61	1.23 ¥
Cox6a2	1.61	6.07	6.70	1.19 ¥ §
Creb3l1	1.40	2.67	1.51	-0.16 §
Cth	1.94	4.82	5.41	2.13 ¥
Cxadr	3.92	4.93	4.54	4.24
Cxcr4	2.31	3.86	3.69	1.73 §
Cyb5r1	3.28	7.03	6.17	3.30
Ddr2	1.85	3.78	3.82	1.84 ¥ §
Dll3	3.43	2.09	3.18	3.53 §
Dlx2	-1.76	-0.07	-1.83	-0.61
Eda	0.65	2.20	1.81	0.46 §
Eif4ebp1	5.63	7.10	8.14	6.35 ¥
Ero1l	3.69	5.40	5.29	3.51 §
Fads3	1.28	2.63	2.47	1.34
Fam13b	2.52	3.72	3.17	2.27 §
Fam129a	-2.29	0.06	-1.37	-1.92
Fbln5	-1.96	0.62	1.32	-1.88 ¥
Fbln7	1.25	3.51	2.93	0.67 §
Fgfr3	5.66	4.69	5.06	5.40
Gas1	4.96	6.01	6.05	5.39 ¥
Gbp2	0.36	2.76	1.78	0.40
Gbp3	2.81	4.38	3.49	2.33 §
Glce	2.79	4.09	4.27	3.11 ¥
Gpr17	4.12	2.52	3.80	3.74
Gpr37l1	-1.96	1.38	2.35	2.51
Gpx3	3.46	2.34	2.41	2.51

Gene	XX Baseline	XY Baseline	XX+TP	XY+TP
Gsg1l	0.44	-1.03	-1.85	-1.42 ¥
Hist1h1c	4.11	6.23	8.61	8.42 ¥
Hlx	1.90	3.15	3.07	1.72 §
Hrk	-1.84	0.43	-0.02	-1.65
Hsd17b1	-1.40	1.36	2.32	-1.07 ¥
Il6ra	-2.98	-0.53	-1.76	-2.94
Jag1	3.59	4.74	4.96	3.83 ¥
Kdm5d	-Inf	2.95	-Inf	3.30
Klf4	2.09	4.00	4.21	2.13 ¥
Lars	4.80	5.91	6.42	5.11 ¥
Ldhb	7.68	6.96	6.98	7.02
Lhfpl2	1.80	3.92	4.12	3.13 ¥
Mei4	-3.63	-1.03	0.21	-2.48 ¥
Mmd2	4.90	3.45	4.54	4.66 §
Mmp28	-2.52	1.22	0.23	-2.78 §
Mthfd2	5.19	6.36	6.90	5.40 ¥
Myh8	-1.08	-3.02	-2.08	-2.16
Nars	6.29	7.42	7.66	6.42 ¥
Ndrg2	4.80	3.46	4.55	4.93 ¥
Nr2f2	0.12	-3.28	-3.72	-3.89 ¥
Nupr1	1.99	6.16	6.70	2.43 ¥
Odz4	-1.84	1.54	-0.29	-1.19
Pck2	3.89	5.40	6.49	4.01 ¥
Plekhb1	5.05	4.14	4.40	4.67
Psph	5.05	6.37	7.10	5.34 ¥
Pygm	4.68	3.37	3.68	3.78
Rhbdd1	2.62	3.68	3.90	2.64 ¥
Sars	6.75	7.93	8.96	7.32 ¥
Scrg1	5.12	3.61	4.60	4.07
Sema4a	-1.03	-2.86	-3.21	-1.72 ¥
Sesn2	5.29	6.31	6.46	5.16 ¥ §
Slc16a14	-1.87	0.02	0.07	-2.31 ¥ §
Slc3a2	6.24	7.80	8.11	6.08 ¥ §
Slc7a1	2.61	5.71	3.91	4.34
Slc7a11	2.61	3.71	3.91	2.27 ¥ §
Slc7a3	2.09	4.82	4.55	2.45
Snhg12	5.12	6.87	6.95	5.25 ¥
Sostdc1	-1.61	0.80	-0.12	-2.11 §
Sv2a	3.32	2.24	2.39	2.32
Tgif1	3.01	4.20	4.38	3.17 ¥
Thrsp	5.25	4.00	4.33	4.43
Thy1	4.91	3.74	0.72	2.61 ¥
Tmem28	-1.08	1.27	-0.39	-1.78 §
Tnnt1	3.45	1.73	2.12	2.19
Trib3	0.71	5.08	5.02	0.75
Ube2ql1	2.37	3.82	3.97	3.10 ¥
Ube1y1	-Inf	-0.44	-Inf	-0.86
Usp18	-2.31	0.18	-2.10	-2.51 §
Uty	-Inf	3.18	-Inf	3.54
Vegfa	4.60	3.93	6.59	4.48
Wars	-1.61	0.80	-0.12	-2.11 §
Xist	6.97	-4.49	5.67	-9.97

¥- Genes that were completely masculinized on a XX background
§- Genes that were completely feminized on a XY background

Supplemental Table 1. The log_2 FPKM values for the 103 genes that were differentially expressed between XX and XY eNSCs (FDR =0.10) in addition to the log_2 FPKM values for these select genes after being exposed to testosterone propionate.

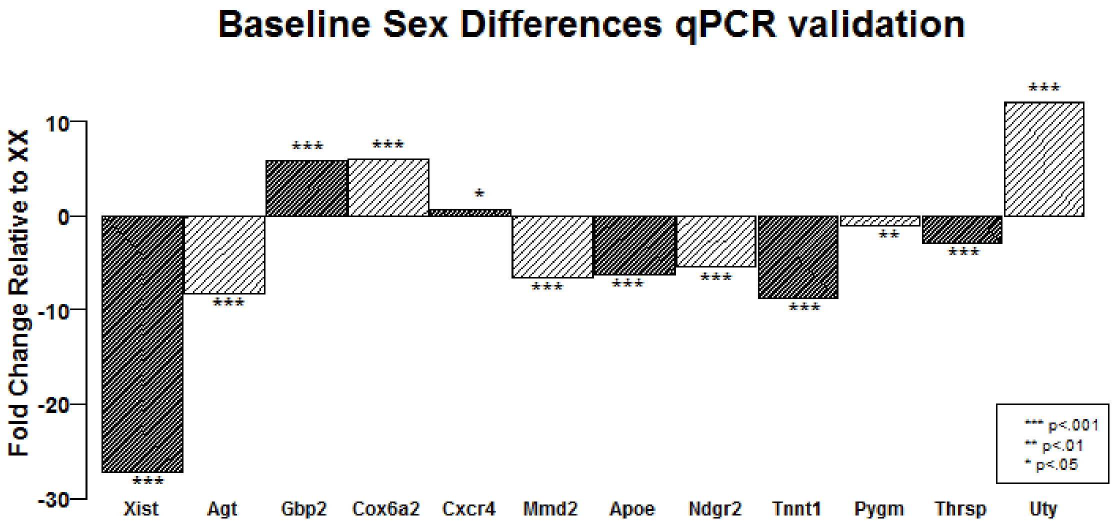

Supplemental Fig 1. qPCR validation of a select set of genes that were found to be differentially expressed between XX and XY neural stem cells within the RNA-seq dataset. Gene expression differences are represented as XY fold change from baseline XX expression.

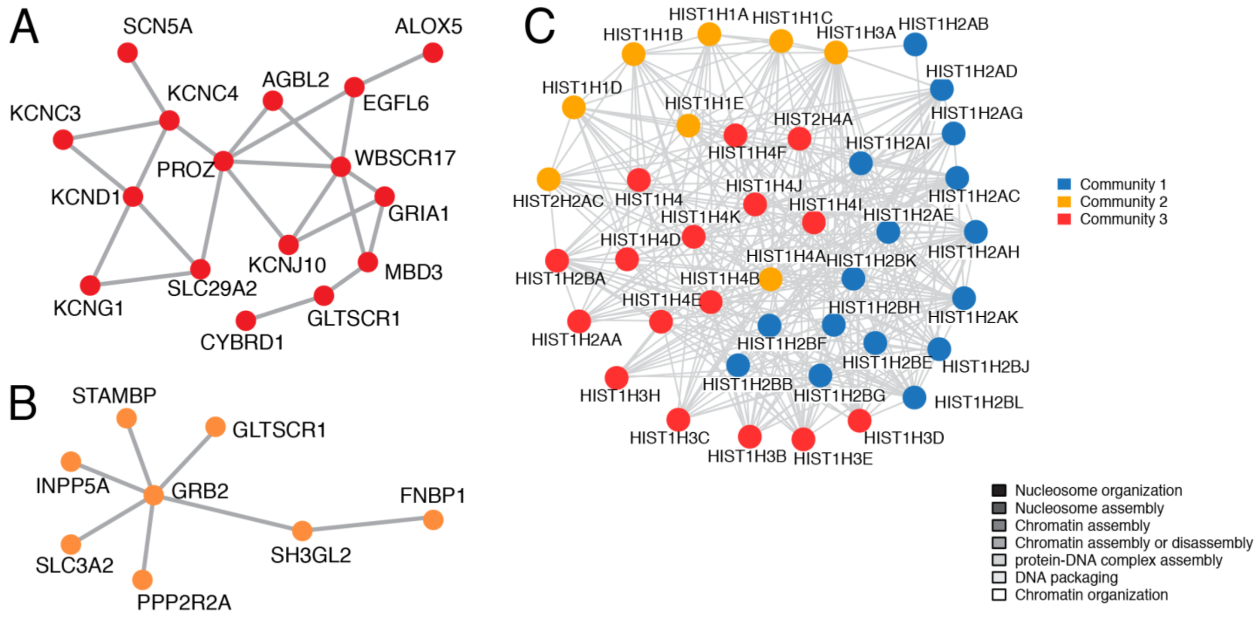

Supplemental Fig. 2: A) Down-regulated genes on an XX background in the presence of TP that are enriched in pathways dealing with potassium channel signaling. B) Down-regulated genes on an XY background that are enriched for pathways dealing with ERRB1 internalization. C) Up-regulated genes that are mutually shared between XX and XY in the presence of TP. The top three most up-regulated pathways are involved with nucleosome organization, nucleosome assembly, and chromatin assembly.

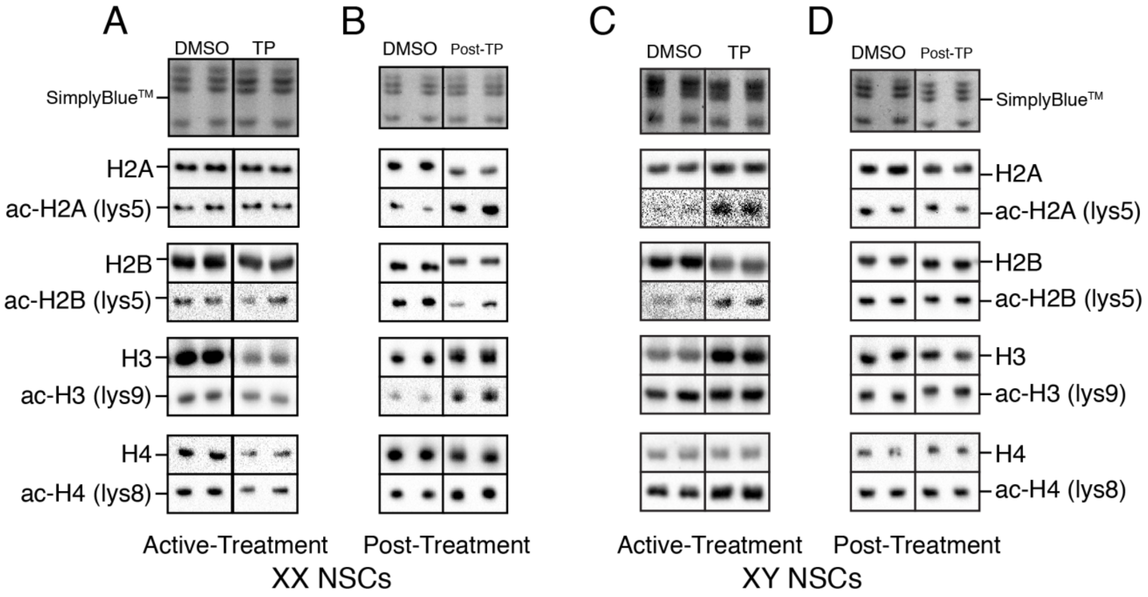

Supplemental Fig 3: A) Representative images of total protein assessment using Simply Blue stain which was used as the initial standardization for protein loading. Representative Western blots for XX and XX in TP measuring both core histone abundance and acetylation of specific residues. B) The same as panel A, except XY and XY in TP is being assayed. C) Representative image of total protein assessment using Simply Blue stain which was used as the initial standardization for protein loading. Representative Western blots for XX daughter cells post DMOS exposure and XX daughter cells post-TP exposure measuring both core histone abundance and acetylation of specific residues. D) The same as panel C, except measuring XY daughter cells post DMSO exposure and XY daughter cells post-TP exposure.
